# Channel-Dependent Multilayer EEG Time-Frequency Representations Combined with Transfer Learning-Based Deep CNN Framework for Few-Channel MI EEG Classification

**DOI:** 10.3390/bioengineering12060645

**Published:** 2025-06-12

**Authors:** Ziang Liu, Kang Fan, Qin Gu, Yaduan Ruan

**Affiliations:** Department of Critical Care Medicine, Nanjing Drum Tower Hospital, Affiliated Hospital of Medical School, Nanjing University, Nanjing 210028, China; ziangliu@smail.nju.edu.cn (Z.L.); kangfan@smail.nju.edu.cn (K.F.); icuguqin@nju.edu.cn (Q.G.)

**Keywords:** few-channel EEG, motor imagery, transfer learning, wavelet transform

## Abstract

The study of electroencephalogram (EEG) signals is crucial for understanding brain function and has extensive applications in clinical diagnosis, neuroscience, and brain–computer interface technology. This paper addresses the challenge of recognizing motor imagery EEG signals with few channels, which is essential for portable and real-time applications. A novel framework is proposed that applies a continuous wavelet transform to convert time-domain EEG signals into two-dimensional time-frequency representations. These images are then concatenated into channel-dependent multilayer EEG time-frequency representations (CDML-EEG-TFR), incorporating multidimensional information of time, frequency, and channels, allowing for a more comprehensive and enriched brain representation under the constraint of few channels. By adopting a deep convolutional neural network with EfficientNet as the backbone and utilizing pre-trained weights from natural image datasets for transfer learning, the framework can simultaneously learn temporal, spatial, and channel features embedded in the CDML-EEG-TFR. Moreover, the transfer learning strategy effectively addresses the issue of data sparsity in the context of a few channels. Our approach enhances the classification accuracy of motor imagery EEG signals in few-channel scenarios. Experimental results on the BCI Competition IV 2b dataset show a significant improvement in classification accuracy, reaching 80.21%. This study highlights the potential of CDML-EEG-TFR and the EfficientNet-based transfer learning strategy in few-channel EEG signal classification, laying a foundation for practical applications and further research in medical and sports fields.

## 1. Introduction

The electroencephalography (EEG) is a non-invasive electrical signal obtained by measuring the activity of neurons in the brain on the scalp surface [1]. EEG reflects the brain’s activity states, including electrical activities at different frequencies (such as α, β, and δ waves), and changes in response to specific cognitive tasks or stimuli, making it widely used in clinical diagnosis [2], neuroscience research [3], and brain–computer interface (BCI) technology [4,5]. Studying EEG signals is crucial for understanding brain functions and applying them in clinical settings.

The study of motor imagery (MI) EEG signals is of significant importance in understanding the neural mechanisms of motor control, rehabilitation medicine, BCI technologies, and motor skill improvement. By analyzing the electrical activities generated by the brain during MI processes, it is possible to delve deeper into the neural basis of motor control, provide new methods for rehabilitation medicine, drive BCI technology development, and assist athletes in improving motor skills. These studies contribute to the in-depth exploration of scientific theories and bring about rich possibilities for practical applications.

In recent years, deep learning (DL) has made significant advancements in the field of EEG signal processing and classification [6]. DL algorithms, such as convolutional neural networks (CNNs) [7], graph convolutional networks (GCNs) [8], recurrent neural networks (RNNs) [9], and long short-term memory networks (LSTMs) [10], have been widely applied to classify and analyze EEG signals. These algorithms extract rich spatio-temporal features from EEG signals and capture dynamic changes in brain activity. For instance, CNNs are effective in learning spatial features at different frequencies, GCNs model the correlations between different brain regions, and RNNs/LSTMs handle the temporal dynamics of EEG signals. The use of these DL algorithms has greatly improved the accuracy and efficiency of EEG signal analysis, supporting the development of BCI technology.

So far, many DL-based EEG classification technologies have been developed, which can be divided into two categories—multi-channel EEG signal classification and few-channel EEG signal classification. The first category is multi-channel EEG signal classification based on a large number of channels. Zhou et al. [11] proposed a novel Lightweight Multi-Attention Network, which can effectively extract the spatio-temporal features of multi-channel EEG signals, achieving high-precision real-time detection of neonatal seizures. Dang et al. [12] regarded each EEG channel as a node and constructed a complex brain network consisting of 23 channels for epilepsy signal classification. Yue Gao et al. [8] proposed a spatio-temporal adaptive graph convolutional network, capable of adaptively capturing important channel positional information in multi-channel EEG for emotion recognition. Jia et al. [13] introduced a novel deep graph neural network capable of learning intrinsic connections between multi-channel EEG signals for automatic sleep stage classification. However, these methods rely more on the multi-channel characteristics of EEG signals, posing challenges in their application to few-channel EEG signals. The second category is few-channel EEG signal classification, which involves fewer channels (e.g., 1–3 channels). In recent years, the development of portable EEG recording hardware and improvements in EEG processing techniques have spurred the emergence of new EEG applications in daily life, such as real-time stress level monitoring [14,15] and MI [16,17]. Meanwhile, the application of few-channel EEG has enhanced portability and wearability, reduced computational load, and accelerated feedback speeds, making it popular in these applications. However, EEG signals with few channels reduce the amount of information, and many methods suitable for the classification of multi-channel EEG signals are difficult to use in scenes with few channels. Therefore, studying EEG signal recognition methods suitable for a few channels is necessary.

Zhang et al. [18] proposed a rapid adaptive sub-band blind source separation method for effectively removing artifacts from short-term, few-channel EEG recordings. Zahra Khademi et al. [19] investigated single-channel classification of EEG signals in MI-based BCIs using three hybrid models. However, their focus was on analyzing the time-frequency characteristics of individual EEG channels and did not consider methods for fusing information from few-channel EEG signals. Xu et al. [20] proposed a method for fusing three-channel EEG time-frequency graphs on a plane for few-channel MI EEG classification, but this method is not scalable enough and fails to effectively utilize the advantages of DL models. Ali Al-Saeghr [21] proposed a novel augmentation method to enlarge EEG datasets, addressing the issue of limited data in few-channel EEG signals. Effectively integrating information from few-channel EEG signals and leveraging the advantages of DL models to improve classification accuracy remains an unresolved issue.

**Figure 1:**
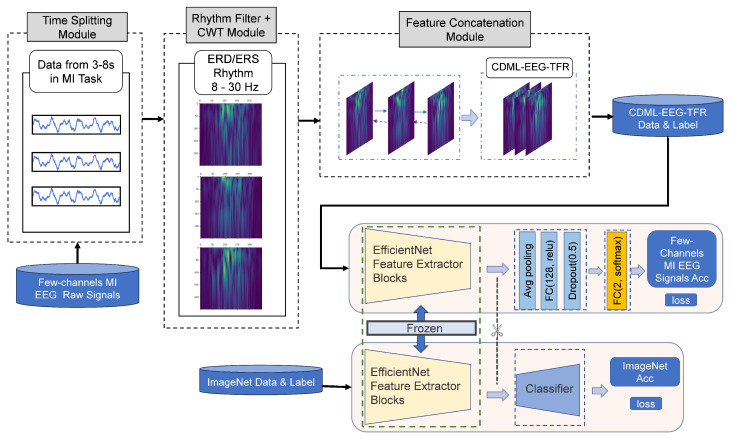
The proposed channel-dependent multilayer EEG time-frequency representation (CDML-EEG-TFR) combined with transfer learning-based deep CNN framework. The original few-channel MI EEG signals are first processed by the time splitting module to extract signal segments related to the MI process (i.e., the third to eighth seconds of each trial), removing other signals from non-MI periods. These segments are then fed into the rhythm filter and continuous wavelet transform (CWT) module for 8–30Hz bandpass filtering, and each channel’s signal is converted into a 2D time-frequency map via CWT. Next, the feature concatenation module integrates the time-frequency maps from different channels along the direction perpendicular to the time-frequency map plane to construct the CDML-EEG-TFR. Finally, the CDML-EEG-TFR is input into an EfficientNet model pre-trained on the ImageNet dataset with frozen parameters, and classification of left/right hand MI is performed via a dedicated classifier.

Few-channel EEG signals suffer from limited spatial resolution and data sparsity due to the restricted number of electrodes, leading to challenges in achieving satisfactory classification accuracy. To address this issue, this paper proposes a novel classification method based on channel-dependent multilayer EEG time-frequency representation (CDML-EEG-TFR) and transfer learning, specifically designed for few-channel EEG classification tasks, as illustrated in Figure 1. The proposed method demonstrates superior classification performance under the challenges of limited spatial information and data sparsity in few-channel EEG signals. The main contributions include the following:

- Designs a novel feature representation for few-channel EEG—channel-dependent multilayer EEG time-frequency representation;
- Validates the effectiveness of transfer learning in addressing data sparsity in few-channel EEG;
- Proves EfficientNet’s effectiveness in the field of EEG signal classification;
- Achieves significant performance improvement on the publicly available dataset.

The rest of this paper is structured as follows: Section 2 (Materials and Methods) introduces the proposed innovative few-channel electroencephalogram (EEG) signal classification method, elaborating in detail on the dataset used, the design principles of the wavelet transform and feature concatenation module, and the transfer learning module. Section 3 (Results) validates the effectiveness of the method through rigorous comparative experiments and ablation studies. Section 4 (Discussion) evaluates the impacts of channel reduction, transfer learning architectures, and different backbone networks on classification performance. Finally, the conclusion section summarizes the core contributions.

## 2. Materials and Methods

This paper introduces a novel framework for few-channel MI EEG signal classification, namely, the CDML-EEG-TFR combined with a transfer learning-based deep CNN framework, as illustrated in Figure 1. The entire framework consists of two components: the CDML-EEG-TFR generation module and the transfer learning module.

**Figure 2:**
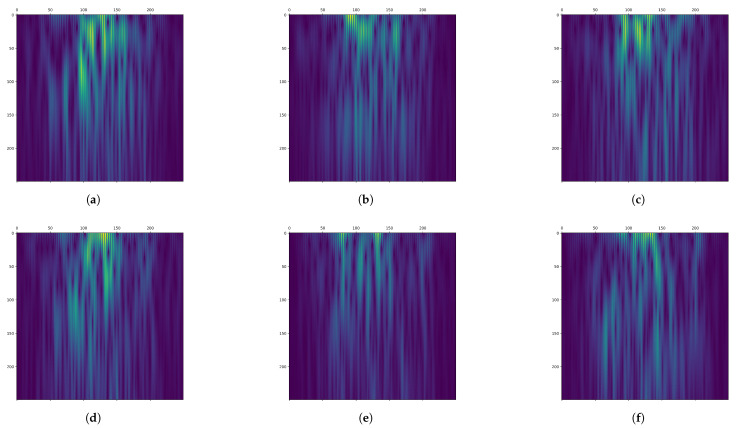
Time-frequency maps of channels (**a**) C3, (**b**) Cz, and (**c**) C4 for left hand, and (**d**) C3, (**e**) Cz, and (**f**) C4 for right hand during a segment of the MI task in the BCI Competition IV dataset 2b after filtering and CWT. Each map has a size of 250 × 250 pixels. The horizontal axis represents time, ranging from the 3rd to 8th s during the MI process with a resolution of 0.002 s; the vertical axis represents frequency, ranging from 8 to 30 Hz with a resolution of 0.088 Hz. Each pixel represents the energy of the signal at the corresponding time and frequency: higher energy corresponds to warmer (yellowish) tones, while lower energy is represented by cooler (bluish) tones.

The CDML-EEG-TFR generation module is divided into three sub-modules: the time splitting module, the rhythm filtering and continuous wavelet transform (CWT) module, and the feature concatenation module. First, raw few-channel EEG signals are fed into the time segmentation module to extract time segments relevant to the motor imagery process and remove invalid portions. Subsequently, the filtering and CWT module performs 8–30 Hz bandpass filtering on the time-domain signals of each time segment in each channel to eliminate noise and artifacts. The filtered signals are then converted into two-dimensional time-frequency images via CWT, where the horizontal axis represents time and the vertical axis represents frequency (as shown in Figure 2), enabling the identification and localization of event-related desynchronization/synchronization (ERD/ERS) phenomena [22,23]. Finally, the feature concatenation module concatenates the time-frequency maps from different channels along the direction perpendicular to the image plane, forming a three-dimensional comprehensive and enriched feature representation termed CDML-EEG-TFR. This representation encapsulates multi-dimensional information, including temporal, spectral, and channel-specific details, enabling in-depth characterization of brain states.

A deep learning network based on transfer learning is designed using EfficientNet [24] as the backbone. First, EfficientNet is pre-trained on the large-scale ImageNet dataset to obtain initial weights. Its original classification head is then removed, and a new classifier is appended, consisting of a global average pooling layer, a fully connected layer with 128 neurons, a Dropout layer with a dropout rate of 0.5, and a final fully connected layer with two neurons using softmax activation. During training and testing, the pre-trained weights of EfficientNet are kept frozen. By leveraging knowledge acquired from natural image datasets, the network is able to extract features from the different channels of few-channel EEG signals, analogous to how color channel features are extracted from natural images. It simultaneously learns temporal, spectral, and inter-channel features embedded in the CDML-EEG-TFR representation. Moreover, the transfer learning strategy effectively alleviates the challenge of data sparsity in few-channel EEG scenarios, enhancing the model’s generalization ability.

### 2.1. Dataset

The performance of the proposed framework was evaluated using data from the BCI Competition IV dataset 2b [25]. The BCI IV dataset 2b consists of EEG data from nine subjects performing two types of MI tasks: imagining left-hand and right-hand movement. In the experiments, EEG signals were recorded using three electrodes (C3, Cz, and C4). All collected signals were band-pass filtered (0.5 to 100 Hz) and a notch filter at 50 Hz, with a sampling frequency of 250 Hz. The dataset comprises five sessions, with the first two sessions containing EEG data from MI tasks without feedback, the third subset containing EEG data from MI tasks with feedback, and the last two sessions containing estimate data. The feedback experimental data from nine subjects were used.

**Figure 3:**
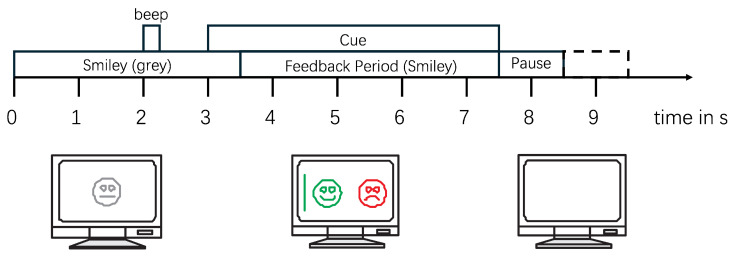
Experimental procedure with feedback for dataset BCI Competition IV 2b [25]. At the beginning of each trial, a gray smiley face is displayed on the computer screen. After 2 s, a 70 ms beep sounds as a prompt for task onset. The MI task is executed from the 3rd to the 7.5th s. At the 3-s mark, visual cues corresponding to the MI task category (left/right hand) are presented. Participants are instructed to imagine moving the smiley face horizontally according to the cues via left/right hand MI. Visual feedback is provided in real time: a green smiley face indicates correct movement direction, whereas a red frustrated face signals an error. At 7.5 s, the screen is cleared, followed by a random inter-trial rest interval of 1–2 s before the next trial begins.

In the experiments with feedback, each subject performed a total of 160 trials (80 trials for each MI task). Each trial lasts for 8–9 s, with the MI task occurring from 3 to 7.5 s. Following this, there is a random interval of 1–2 s as rest time between different test trials, as illustrated in Figure 3. To preserve the complete information of the MI process and eliminate interference from non-MI EEG signals, 5 s of data starting from the onset of each MI task (from the 3rd to the 8th s as shown in Figure 3) for each trial of each subject was extracted, resulting in a total of 1440 samples for all nine subjects, with a duration of 7200 s. All of these samples constituted the dataset used in this study.

### 2.2. Wavelet Transform and Feature Concatenation Module

During MI tasks, EEG signals exhibit characteristic changes: a decrease in amplitude in the μ band (8–13 Hz), known as event-related desynchronization (ERD), and an increase in amplitude in the β band (13–30 Hz), known as event-related synchronization (ERS). To retain the essential frequency components while minimizing noise and artifacts, the raw EEG signals were preprocessed using a second-order Butterworth band-pass filter ranging from 8 to 30 Hz.

Based on the neurophysiological mechanisms of the sensorimotor cortex during MI tasks, distinct electrophysiological changes occur in the contralateral sensorimotor areas when imagining left- or right-hand movements. The C3 and C4 electrodes, corresponding to the left and right hemispheric sensorimotor cortices, respectively, are strategically positioned to capture ERD phenomena in the contralateral brain regions during limb movement imagination. The Cz electrode, located along the central midline, monitors coordinated bilateral motor activity. Collectively, these channels comprehensively cover the core response regions critical to MI task execution. It was found in [26,27] that EEG signals from electrodes C3, C4, and Cz are significantly influenced during motor intention tasks. Consequently, the C3, Cz, and C4 channels were selected as the primary electrophysiological targets in this investigation.

Expressing features only in the time domain and ignoring frequency domain information may degrade classification accuracy. A previous study [28] demonstrated that combining the frequency and time characteristics of EEG signals can effectively improve classification results. Therefore, it is necessary to span the representation of features into the two-dimensional time-frequency domain.

EEG signals are highly non-stationary and contain multiple spectra [29]. The wavelet transform constructs a time-frequency domain signal with precise time and frequency localization. The frequency components contained in the EEG signal and their corresponding time segments can be observed to identify the occurrence and localization of the ERD/ERS phenomenon. Among the various methods of wavelet transform, CWT can effectively process non-stationary single-channel EEG signals and avoids the issue of window size selection encountered in Short-Time Fourier Transform methods [30]. CWT is a commonly used tool for time-frequency analysis, allowing the localized analysis of signals in both temporal and spectral domains. It achieves this by filtering signals at different scales and shifting filters in time, resulting in a time-frequency representation of the signal.

The principle of CWT is based on smoothing the signal at different scales and frequencies. This is achieved by convolving the signal with a set of continuous wavelet functions, typically obtained by scaling and shifting a mother wavelet function. The mathematical representation of CWT is as follows:

(1) $$W\left(s,t\right)=\frac{1}{\sqrt{s}}\int x\left(t\right)\varphi^{*}\left(\frac{t-\tau }{s}\right)dt$$

where x(t) is the input time series, *s* is the scale parameter of the wavelet transform, φ is the wavelet basis function, and τ is the time shift.

In selecting wavelet base functions, smooth and continuous sequences are typically desired after performing CWT on EEG signals; thus, non-orthogonal wavelet functions should be chosen. Morlet wavelets are often used for EEG signal analysis due to their non-orthogonality and good balance between time and frequency localization. Compared with other commonly used wavelet families such as Haar and Daubechies, which are more suitable for piecewise smooth signals and tend to introduce discontinuities, the Morlet wavelet provides better frequency resolution due to its Gaussian-modulated sinusoidal form. This makes it particularly effective for capturing the rhythmic oscillatory patterns present in EEG signals. Additionally, the Morlet wavelet’s continuous and symmetric shape enables smoother and more interpretable time-frequency maps, which are beneficial for subsequent classification tasks. Comparisons have shown that EEG signals based on Morlet wavelet transform achieve optimal classification results [31]. Therefore, the Morlet wavelet was chosen as the wavelet base function. The formula for the Morlet wavelet is as follows:

(2) $$\Psi_{\tau ,s}\left(t\right)=\frac{1}{\sqrt{s}}\pi^{-1/4}e^{i\omega_{0}\frac{t-\tau }{s}}e^{-\frac{1}{2}{\left(\frac{t-\tau }{s}\right)}^{2}}$$

where *s* is the scale parameter of the transform, and τ is the time shift.

To better capture the frequency bands (8–30 Hz) essential for MI tasks, we adapted the scale range of the CWT according to the target frequencies and signal sampling rate.

(3) $$s_{\min}=\frac{f_{c}f_{s}}{f_{\max}}$$

(4) $$s_{\max}=\frac{f_{c}f_{s}}{f_{\min}}$$

where $s_{\min}$ and $s_{\max}$ are the minimum and maximum scale parameters of the transform, $f_{c}$ is the center frequency, $f_{s}$ is the sampling frequency, and $f_{\min}$, $f_{\max}$ represent the minimum and maximum frequencies of the target frequency band.

The output of CWT is a two-dimensional matrix, with one dimension representing time and the other representing scale. On the time-scale plane, CWT reveals the local frequency information of the EEG signal. Figure 2 shows the time-frequency maps of channels C3, Cz, and C4 from a segment of the MI task in the BCI Competition IV 2b dataset after filtering and CWT transformation. The MI of the left hand is depicted in the first row, while the MI of the right hand is depicted in the second row.

From Figure 2, it can be observed that the time-frequency representation exhibits a flame-like pattern, with the low-frequency region appearing brighter. A horizontal comparison of the time-frequency representations across different channels reveals variations in the distribution of the highlighted regions. A vertical comparison of different MI tasks for the same channel shows that the flame is brighter for the MI of the left hand compared with the MI of the right hand. Therefore, different MI tasks correspond to significant differences in EEG signals across time-frequency and channel domains. The time-frequency representation effectively captures these differences in time-frequency characteristics.

However, using CWT alone may not fully capture the channel characteristics of few-channel EEG signals. Therefore, inspired by the input format of color images with RGB channels, we applied a feature concatenation approach to connect time-frequency map features from different channels, forming a channel-dependent multi-layered EEG time-frequency representation with a comprehensive and rich feature set. As shown in the feature concatenation module of Figure 1, the time-frequency maps of the C3, C4, and Cz channels were analogized to the R, G, and B channels of color images and stacked along the direction perpendicular to the frequency-time plane of the image to create a multi-layered three-dimensional EEG time-frequency representation, where the three dimensions represent time, frequency, and channel, respectively. This effectively combines the temporal, spectral, and channel features of EEG signals, establishes a cross-channel joint representation space, and fully characterizes brain states under the constraint of a limited number of channels. Additionally, its dimensional structure is compatible with the input dimensions of standard CNNs. When input into CNNs, the convolution kernel can synchronously extract three complementary feature modalities in a single operation: temporal dynamics (sliding along the time axis), frequency-domain features (convolution along the frequency axis), and topological relationships between channels (weight allocation along the channel axis). This enables CNNs to more effectively utilize the spatial and channel feature extraction capabilities learned from natural images to mine the time-frequency domain features of EEG signals and the associative features between different EEG channels.

### 2.3. Transfer Learning Module

The obtained CDML-EEG-TFR was used as input for the DL network based on transfer learning for few-channel MI EEG signal classification.

Several CNNs have been pre-trained on large-scale datasets, including AlexNet [32], VGGNet [33], Inception [34], ResNet [35], and EfficientNet, among the most popular pre-trained networks. They have been trained on the ILSVRC dataset [36], a subset of the ImageNet dataset comprising 1.2 million images from 1000 different categories, covering animals and objects. Since the weights of pre-trained networks can adapt well to large-scale image sets, they can be used for feature extraction, resulting in better performance compared with traditional CNN networks with random weights.

EfficientNet, an efficient and accurate convolutional neural network architecture proposed by the Google research team in 2019, achieves high performance under limited computational resources by using compound scaling, which increases network depth, width, and resolution while keeping the model’s parameter count and computational complexity relatively low. The core idea of EfficientNet is to maintain efficiency while increasing model size. Its compound scaling involves scaling three factors: network depth, network width, and input resolution. Through this compound scaling approach, EfficientNet effectively improves model performance without significantly increasing parameters and computational costs. EfficientNet benefits from its innovative architecture and compound scaling. Compared with other pre-trained networks, EfficientNet maintains model accuracy while having a smaller model size and lower computational complexity. EfficientNet outperforms other networks in terms of parameter efficiency, generalization ability, and computational cost through compound scaling strategies, lightweight module design, and dynamic training mechanisms. It is particularly suitable for scenarios with limited data scale and exhibits excellent transferability. Therefore, we applied EfficientNet as the backbone for EEG signal classification experiments, demonstrating its feasibility in the field of EEG signal classification. Among the various sizes of EfficientNet models, we selected the smallest one, EfficientNet-B0, due to its reduced number of parameters and faster inference speed, making it more suitable for real-time detection in few-channel EEG monitoring.

We pioneered the use of natural image knowledge learned by EfficientNet to guide EEG classification and validate the effectiveness of this transfer learning approach. In scenarios with few-channel EEG signals, the small data sparsity hinders deep learning networks from accurately extracting data features and often leads to overfitting. To address this issue, transfer learning by leveraging the pre-trained weights of EfficientNet on the ImageNet dataset was applied. Although there is a large domain discrepancy between EEG signals and natural images, we alleviate this issue by transforming EEG time-domain signals into CDML-EEG-TFR. Each layer of the time-frequency representation resembles one channel in an RGB image, effectively constructing a multi-channel image-like representation. This structure captures the temporal, spectral, and spatial (channel) features of EEG signals in a way that aligns with the format expected by pre-trained CNNs. As a result, the transfer learning strategy can be more effectively applied, enabling the network to leverage features learned from natural image datasets. The CDML-EEG-TFR ingeniously integrates the temporal, frequency, and channel features of EEG signals into a format resembling a three-channel color image. This allows EfficientNet, which has learned from natural image datasets, to effectively extract the temporal, spectral, and channel features from CDML-EEG-TFR, thereby mitigating the problem of limited dataset size.

To specialize these pre-trained networks for our classification purposes, all fully connected layers were removed. These fully connected layers were designed for the original tasks (such as ImageNet classification), so they were redesigned for our task. Then, a global average pooling layer was added, which took the average of all values of each channel of the convolutional feature maps, resulting in a fixed-size feature vector independent of the number of channels. Next, a fully connected layer with 128 neurons was added, using ReLU as the activation function, to map the feature vector output from the pooling layer to a higher-dimensional feature space. To reduce the risk of overfitting, a Dropout layer with a dropout rate set to 0.5 was added after the fully connected layer to enhance the model’s generalization ability. Finally, a fully connected layer with two neurons, using softmax as the activation function, served as the final classifier.

## 3. Results

### MI Classification Result

Taking subject 1 as an example, two randomly selected CDML-EEG-TFRs are illustrated in Figure 2. Each subplot corresponds to a layer of the CDML-EEG-TFR, associated with a specific EEG channel. The MI of the left hand is depicted in the first row, while the MI of the right hand is depicted in the second row. It can be observed that the time-frequency maps exhibit significant differences across different channels and brain MI states. These findings indicate that the proposed CDML-EEG-TFR can effectively characterize brain changes during the MI of left and right hand movements, confirming the importance of time, frequency, and electrodes (channels) in MI detection research.

MI-EEG data were tested for classification using ten-fold cross-validation in all experiments. The data of each subject were divided into ten equal, disjoint subsamples of the same size. One was used to test the model, and the other nine were used to train the model. This process was repeated until each aliquot of subsamples was tested (i.e., ten sets of results were obtained), and the average was used as the final average classification rate. In the experiments, the classification accuracy was used to evaluate the performance of the proposed framework. The CDML-EEG-TFRs, fused with multidimensional information of time, frequency, and channels, were utilized as input. They were fed into the pre-trained EfficientNet-B0 model, which had been trained on ImageNet, to accelerate training and achieve better classification results on the small-scale EEG dataset. EfficientNet-B0 was trained using Keras in a fully supervised process. Using the Adam optimizer, various weights and biases were optimized by minimizing the cross-entropy loss function. The best model was recorded when the validation set loss reached its minimum. All procedures were executed on a workstation equipped with an Intel CPU (i7-7700K, 4.2 GHz), NVIDIA GPU (GTX TITAN X), and 16 GB RAM. We adopted the TensorFlow 1.13.0 framework running on the Python 3.7 platform to train and evaluate our proposed network. The learning rate was set to 0.001, and training was conducted for 150 epochs with a batch size of 32.

Accuracy was used as the performance metric to evaluate the model’s effectiveness. The proposed approach performed well on all performance metrics, with an average accuracy of 80.21%. Table 1 presents existing studies on the BCI IV 2b dataset with sample lengths equal to or longer than 0.8 s. Our proposed DL framework, CDML-EEG-TFR, combined with transfer learning, achieved the best results on the BCI IV 2b dataset, demonstrating its excellent representation capabilities for few-channel EEG signals. Additionally, it shows that using weights trained on a natural image dataset for transfer learning can effectively mitigate the information loss inherent in few-channel EEG signals.

In addition to the results shown in Table 1, we also computed the accuracy of the proposed MI detection approach considering only a single channel, and the results are presented in Table 2. To evaluate the effectiveness of utilizing large-scale pre-trained weights, we compared the performance of the EfficientNet-B0 model with and without pre-trained weights on the BCI IV 2b dataset, with the experimental results shown in Table 3. Furthermore, we replaced the backbone of the transfer learning network with ResNet50 and Inception-v3 and compared their performance with EfficientNet-B0. Table 4 presents the experimental results of different transfer learning network backbones under our designed detection framework on the BCI IV 2b dataset.

## 4. Discussion

In this paper, CWT is utilized to transform the time-domain EEG signals into two-dimensional images with time (X-axis) and frequency (Y-axis) as coordinates, referred to as time-frequency maps. Then, a feature concatenation approach is utilized to stack the time-frequency graphs of each channel along the Z-axis. This forms a CDML-EEG-TFR, which effectively integrates the temporal, spectral, and channel features of EEG signals. This enables us to fully leverage inter-channel correlation in few-channel EEG signals, thereby enhancing the performance and stability of the classification system.

This paper creatively applies a transfer learning strategy based on CNNs to tackle the data sparsity issue in few-channel EEG, which can simultaneously extract the temporal, spectral, and channel features hidden in the CDML-EEG-TFR. This strategy leverages prior knowledge from natural image datasets to guide the model in classifying few-channel EEG signals. With the aid of this prior knowledge, the model learns to extract and interpret features from each EEG channel analogously to how it processes color channels in natural images. This strategy alleviates the data sparsity caused by limited EEG channels, significantly improving the model’s learning capability in few-channel scenarios. Additionally, EfficientNet is adopted as the backbone network for EEG signal classification, which, to the best of our knowledge, is the first time it has been applied to EEG data. We proved its effectiveness in the field of EEG signal classification.

Our approach achieved a significant performance improvement, reaching 80.21%, on the publicly available BCI Competition IV 2b dataset. This demonstrates that CDML-EEG-TFR has excellent representation capabilities for few-channel EEG signals. It also shows that using weights trained on a natural image dataset for transfer learning can effectively mitigate the information loss inherent in few-channel EEG signals.

This work provides a new perspective for accurate classification of few-channel EEG signals under resource-constrained conditions, with potential applications in brain–computer interfaces and clinical diagnostics. Currently, our proposed framework has only been applied to a few-channel MI EEG classification. However, this method for feature representation and classification of few-channel EEG signals can be extended to other EEG-based tasks (such as sleep stage classification and seizure detection). The current pipeline, including preprocessing, channel selection, and time-frequency parameterization, is optimized for the MI tasks. Adapting this method to new domains necessitates task-specific adjustments: (1) reselecting channels to capture task-relevant brain activity, (2) optimizing frequency bands and CWT parameters for target spectral signatures, and (3) refining preprocessing pipelines to suppress domain-specific artifacts (e.g., muscle noise in seizure data). In the future, we will explore the application of this framework in other domains. Tests and further improvements on other few-channel EEG-based tasks will also be conducted in the future.

### 4.1. Channel-Dependent Multilayer EEG Time-Frequency Characterization Analysis

Researchers have conducted numerous studies on MI detection, many of which are related to frequency analysis or channel analysis. However, until now, few studies have considered time, frequency, and channels together. We introduced the concept of CDML-EEG-TFR, which is derived from multi-channel EEG signals and contains multidimensional information, including time, frequency, and channels, enabling comprehensive characterization of brain states.

The results, presented in Table 2, indicate that the classification accuracy of CDML-EEG-TFR is significantly higher than that of single-layer time-frequency representations. This demonstrates that CDML-EEG-TFR effectively integrates EEG features and enhances the performance of DL networks in classification tasks. This result also supports the notion that limited spatial information and data sparsity in few-channel EEG can negatively affect MI classification performance. Nevertheless, our proposed CDML-EEG-TFR and transfer learning framework alleviates these challenges and still achieves competitive performance under resource-constrained conditions.

### 4.2. Design for Transfer-Learning

In our experiments, we applied a combination of transfer learning and CDML-EEG-TFR for detecting MI EEG signals with few channels. In the transfer learning approach, we choose to utilize the EfficientNet-B0 network, which maintains accuracy while having a smaller size and lower computational complexity. To facilitate rapid learning, we leveraged the large-scale pre-trained weights of EfficientNet-B0 on the ImageNet dataset. Adapting these pre-trained weights, originally designed for general image classification, to EEG signal classification is challenging due to significant differences between the source domain (ImageNet images) and the target domain (EEG time-frequency representations). The experimental results (as shown in Table 3) indicate that the EfficientNet-B0 model using pre-trained weights achieved an accuracy of 80.21% on the BCI IV 2b dataset, while the model without pre-trained weights achieved an accuracy of 72.50%. This demonstrates that utilizing pre-trained weights from large-scale general image datasets for MI signal classification with few channels is feasible and can lead to higher accuracy.

### 4.3. Comparison of Backbones

Compared to other pre-trained networks, EfficientNet-B0 maintains model accuracy while having a smaller model size and lower computational complexity. In this paper, we first utilized EfficientNet-B0 as the benchmark model for MI signal classification experiments to verify its effectiveness in classifying MI EEG signals. The experimental results (as shown in Table 4) indicate that EfficientNet-B0 achieved the highest detection accuracy at 80.21%, while ResNet50 and Inception-v3 achieved accuracies of 74.31% and 75.69%, respectively. These findings demonstrate that combining CDML-EEG-TFR with transfer learning is feasible for classifying MI EEG signals with few channels. It also suggests that the adopted EfficientNet-B0 model is practical and effective for classifying MI EEG signals with few channels. In practical applications based on wearable devices, our proposed method can acquire EEG signals from MI subjects and establish an auxiliary system. The high accuracy detection performance can provide timely feedback to subjects on their performance outcomes, while the few-channel detection scheme can make their experiments more convenient and comfortable.

## 5. Conclusions

With the development and emergence of portable EEG recording hardware in daily life, research on methods for recognizing MI EEG signals using few channels has received widespread attention worldwide.

In this paper, we proposed a novel framework for classifying MI EEG signals based on few channels, using a CDML-EEG-TFR fusion deep CNN based on transfer learning. The CDML-EEG-TFR is obtained from few-channel EEG signals through CWT and feature concatenation, containing multidimensional information of time, frequency, and channels, which can comprehensively characterize the brain state. Additionally, this construction enables us to fully exploit inter-channel correlation information in few-channel EEG signals, thereby enhancing the performance and stability of the classification system. The transfer learning strategy effectively addresses the issue of data sparsity in the few-channel scenario. Moreover, we applied the high-performance EfficientNet as the backbone network and utilized pre-trained weights on ImageNet for classifying few-channel EEG signals, demonstrating its effectiveness in this field.

Ultimately, our approach achieved a significant performance improvement on the BCI Competition IV 2b dataset, reaching an accuracy of 80.21%. This demonstrates that CDML-EEG-TFR has excellent representation capabilities for few-channel EEG signals. Additionally, it shows that using weights trained on a natural image dataset for transfer learning can effectively mitigate the information loss inherent in few-channel EEG signals.

In the future, our approach is planned to be tested and improved on more challenging few-channel EEG datasets. This includes exploring robustness under diverse EEG recording conditions, such as varying electrode placements, noise environments, and subject movements. These efforts will not only further validate the effectiveness of our approach but also help adapt it for practical applications under real-world conditions. We also anticipate the broader application of our techniques in practical domains, such as clinical neuroscience and sports science, where reliable and efficient EEG-based brain–computer interfaces can contribute meaningfully to human health, performance monitoring, and well-being.

## Figures and Tables

**Table 1:** MI Classification Accuracies (%) on BCI Competition IV Dataset 2B. The Best Result is Marked in Boldface.

Classfication Acurracy (%)	S1	S2	S3	S4	S5	S6	S7	S8	S9	Avg
Jiao et al. [37]	76.30	56.00	49.20	**98.20**	91.10	74.80	**88.30**	85.40	84.90	78.20
Oikonomou et al. [38]	70.63	56.79	58.44	96.25	**91.25**	81.25	73.44	90.63	86.56	78.34
Al-Saegh et al. [21]	75.31	60.00	**60.31**	97.19	82.81	**82.50**	74.69	88.13	85.00	78.44
Moufassih et al. [39]	69.37	60.00	57.19	95.00	80.63	80.00	80.31	**91.87**	80.63	77.22
Proposed method	**77.50**	**67.50**	55.00	98.13	88.75	74.38	86.88	86.88	**86.88**	**80.21**

**Table 2:** Comparison of Classification Accuracy When Considering Only Single Channel EEG-TFR and Using CDML-EEG-TFR. The Best Result is Marked in Boldface.

Methods	Accuracy (%)
only Channel C3 EEG-TFR	62.50
only Channel Cz EEG-TFR	56.94
only Channel C4 EEG-TFR	65.97
CDML-EEG-TFR	**80.21**

**Table 3:** Ablation Studies on Transfer Learning. The Best Result is Marked in Boldface.

Methods	Accuracy (%)
w/o pre-trained weights	72.50
w/pre-trained weights from the ImageNet dataset	**80.21**

**Table 4:** Comparison of Classification Accuracy Between Different CNN Backbone. The Best Result is Barked in Boldface.

Backbone	Millions of Parameters	Accuracy (%)
ResNet50	23.85	74.31
Inception-v3	22.07	75.69
EfficientNet-B0	4.21	**80.21**

## Data Availability

The data presented in this study are available in BCI Competition IV at https://www.bbci.de/competition/iv/ (accessed on 4 January 2024), reference number [25].

## Abbreviations

The following abbreviations are used in this manuscript:

EEG	electroencephalogram
CDML-EEG-TFR	channel-dependent multilayer EEG time-frequency representations
BCI	brain–computer interface
MI	motor imagery
DL	deep learning
CNNs	convolutional neural networks
GNNs	graph convolutional networks
RNNs	recurrent neural networks
LSTMs	long short-term memory networks
ERS	event-related synchronization
ERD	event-related desynchronization
CWT	continuous wavelet transform
